# Barriers in an East African Refugee Camp: Applying the Three Delays Framework to Pediatric Surgical Care

**DOI:** 10.21203/rs.3.rs-6406297/v1

**Published:** 2025-05-08

**Authors:** Paul Phan, Alexander J. Blum, Matthew D. Price, Mohamed Y. Rafiq, Omar Juma, Frank Manyama, Hilary Ngude, Joseph V. Sakran, Abdulgafoor M. Bachani, Kent A. Stevens, Daniel S. Rhee, Zachary O. Enumah

**Affiliations:** Johns Hopkins University School of Medicine; Brigham and Women’s Hospital; Johns Hopkins University School of Medicine; New York University; Ifakara Health Institute; Tanzania Red Cross Society; Tanzania Red Cross Society; Johns Hopkins University School of Medicine; Johns Hopkins Bloomberg School of Public Health; Johns Hopkins University School of Medicine; Johns Hopkins University School of Medicine; Johns Hopkins University School of Medicine

**Keywords:** Global Surgery, Refugees, Disparities, Three Delays

## Abstract

**Purpose:**

In global health, the three-delays framework helps assess barriers associated with seeking (D1), reaching (D2), and receiving health care (D3). This study applies this model to identify factors contributing to delays in surgical care among children living in a Tanzanian refugee camp.

**Methods:**

A validated surgical needs survey was administered to parents/guardians of children (0–18 years) living in Tanzania’s Nyarugusu refugee camp. We quantified the number of children seeking, reaching, and receiving care for surgically-amendable concerns according to the three-delays framework. Multivariate logistic regression models identified significantly associated predictors with each delay.

**Results:**

721 patient surgically-amendable concerns were identified among 513 children, with 247 children (48.1%) experiencing a delay for at least one problem. Characteristics associated with delays in seeking care included older age, self-perception of good health, and not previously using a primary health center. Burns were also associated with delays in seeking and reaching care, while Congolese nationality and self-perceptions of good health were statistically significant predictors for delays in receiving care.

**Conclusion:**

Children in a Tanzanian refugee camp experience unmet surgical needs complicated by multifactorial delays to seeking, reaching, and receiving surgical care. Future interventions that reduce delays are essential to improve outcomes for refugee children.

## Introduction

Advances in the treatment of communicable diseases have significantly reduced child mortality globally [1]. However, access to surgical care for children remains critically limited: only 3% of children in low-income countries and 8% in middle-income countries have access to adequate surgical services [2]. As a result, many children either die, or live with preventable morbidities that hinder physical development and impose profound psychosocial burdens [2, 3]. In Sub-Saharan Africa, children (0–18 years old) represent half the population and bear roughly one-third of the global burden of surgical disease[1, 4, 5]. Timely access to appropriate surgical care could prevent up to one-third of childhood deaths in this region, highlighting an urgent need for focused interventions in low-to-middle-income countries (LMICs) [6, 7]. Despite meaningful research on barriers to surgical care in LMICs, little is known about the challenges faced by refugee children, who constitute more than half of the global refugee population [8]. Existing studies have primarily examined resettled refugee populations or have not focused on pediatric surgery in humanitarian settings [9–11]. This knowledge gap is concerning, as children living in refugee camps face unique vulnerabilities that exacerbate the burden of surgically-intervenable conditions [12].

The Three Delays Framework—originally developed to understand delays in maternal care—offers a comprehensive approach to evaluating barriers to seeking, reaching, and receiving healthcare [13, 14]. While the framework has been applied to global surgery and pediatric care in Somaliland and other contexts, its use in analyzing surgical delays among children in refugee camps remains understudied [15]. This framework provides a structured lens to better characterize and distinguish the multifaceted barriers refugee children face across the surgical care continuum.

Previously, our work has used data from hospital log books in Nyarugusu to report a greater burden of conditions amendable to surgery among refugee populations compared to non-refugee populations, with nearly 1 in 6 refugee children needing surgical care, such as herniorrhaphies and exploratory laparotomies [3, 16]. Building upon our group’s prior work, this study uses an active surveillance approach to identify barriers and predictors for delays in seeking, reaching, or receiving surgical care for refugee children, including those who have not previously used formal healthcare services in Tanzania, according to the three-delay framework. This research can help inform interventions and policies to improve access to surgical care for children living in refugee camps and humanitarian settings.

## Methods

### Study setting

This study was conducted in the Nyarugusu refugee camp, which is located in the Kigoma region of western Tanzania. The camp has been in continuous operation since 1996 and was home to approximately 150,000 refugees from the Democratic Republic of Congo and Burundi at the time of the study [17]. The camp is organized into 14 zones, which are further divided into villages and clusters of various sizes.

### Data collection

This study is a secondary analysis using previously collected data (August 4, 2021 to September 10, 2021) focused on assessing the burden of surgical disease among refugees in western Tanzania. Surgical conditions were defined using the Lancet criteria as “any disease, illness, or injury in which surgical care can potentially improve the outcome”[16]. Children with health concerns that may have been amenable to surgical intervention were identified through self-reporting by the parents/guardians of the children. For the purpose of this study, seeking care at in-camp physician-based health centers or formal health care centers was considered to be healthcare-seeking behavior. Seeking alternative modalities of healing along the surgical care continuum, including care provided by traditional healers, was coded as a separate variable that could contribute to delays in biomedical management. If healthcare was sought at a clinic or hospital, respondents were asked to specify the type of care received as one of the following: (1) no healthcare or surgery received; (2) healthcare received (i.e. medications) without surgery; (3) a minor surgical procedure performed; or (4) a major surgical procedure performed.

**Figure 1:**
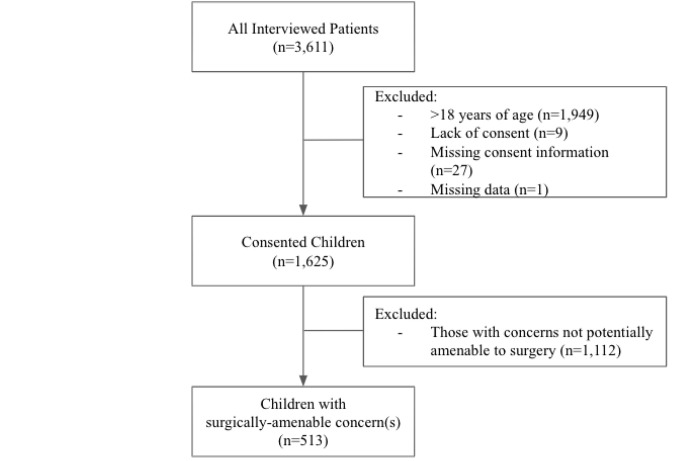


In the parent study, random cluster sampling was used to select 132 clusters out of 1,472, based on the administrative divisions of zones, villages, clusters, and households. Within each cluster, every household was approached; a maximum of two individuals from each household were randomly chosen to participate in the survey. Ultimately, there were 3,611 refugees interviewed across 126 clusters (out of 132 initially selected clusters). Thirty-six records were removed for participants who did not consent (n = 27) or for whom consent was missing (n = 9), and one record was removed for technical reasons (e.g., 99% missing data), yielding 3,574 records that were included (99% response rate). For this particular study, additional exclusion criteria included being over 18 years of age (n = 1,949) and having surgical conditions not amendable to surgery (n = 1,112) (Fig. 1). The Surgeons Overseas Assessment of Surgical Needs (SOSAS) tool was used to assess surgical needs and barriers and was adapted to include questions about referral patterns [18]. This survey tool has been validated and applied in various LMICs and with pediatric and refugee populations [19]. Additional information regarding sampling and power calculation are detailed in the parent study [16].

Data collection was conducted offline using REDCap Mobile and was uploaded to a secure Johns Hopkins University REDCap data server. The data collection process was carried out by refugee community healthcare workers who received training prior to data collection to ensure they could adequately address patient concerns and questions related to cultural differences.

### Applying the Three-Delay Model

Barriers to surgical care for children were reported by survey respondents and included the following options: financial constraints, lack of transportation, fear or lack of trust, perceived unavailability of care, lack of knowledge about available treatment, or perceived lack of need. Respondents could identify multiple health concerns as well as multiple barriers to seeking or receiving care for each concern. The first delay (D1)—delays in seeking care—was defined as not seeking care for any reason including (1) preferential initial consultation of a traditional healer; (2) a perceived lack of need; (3) fear and/or lack of trust in health facilities; (4) financial limitations; (5) lack of knowledge about available treatments; (6) lack of accessible health services; and (7) time constraints. The second delay (D2)—delays in reaching care—was defined as needing more than one hour to be transported to an in-camp health facility and/or waiting more than one hour for transport. A one-hour cutoff was used because the camp’s main hospital is centrally located and readily accessible by foot. The third delay (D3)—delays in receiving care—was defined as needing to wait at least six hours before receiving care after reaching an in-camp health facility (Table 1).

### Data analysis and ethical approval

All statistical analyses were performed using R version 3.0.1 [20]. Descriptive analyses were performed using t-tests and Chi-square tests, in which continuous variables were reported as means with standard deviation and categorical variables were presented as total numbers and relevant percentages. A p-value of less than 0.05 was used to determine statistical significance. Multivariable logistic regression was used to assess for associations between specific covariates and the presence of a delay in seeking, reaching, and/or receiving surgical care. Odds ratios and 95% confidence intervals are reported with standard errors adjusted for the 126 clusters in the final analysis. For multivariable models, age, sex, nationality, literacy status, current health status, primary health center (PHC) utilization within the past year, pathology of concern (e.g. burns, wounds, congenital deformities, masses, etc.), and illness within the past year were included. Camp zone, education, religion, occupation, and/or marital status were not used due to a high degree of collinearity with other predictor variables or lack of sufficient responses resulting in a highly inflated standard error. Additionally, because participants who had previously used a PHC did not have transportation times available to classify whether they experienced delays in reaching or receiving care, the prior use of a PHC was not included in the multivariable models.

The study was approved by the Johns Hopkins Medicine Institutional Review Board (IRB00258009). Research clearance was also obtained from the Tanzanian Commission on Science and Technology (2020–391- NA-2011–143). A permit to enter the refugee camp was granted by the Tanzanian Ministry of Home Affairs. Informed consent/assent was obtained from all participants/their parents/guardians/adult members of the household.

## Results

### Demographics

In this study, 513 children with a combined total of 721 health concerns were included. The median age of the sample population was 11.0 years [IQR: 6.0–16.0], and the most populous age bracket was children aged 12 to 18 years (n = 236, 46.0%), followed by those aged 6 to 11 (n = 140, 27.3%) (Table 2). A slight majority of the participants were male (n = 272 (53.0%)). There were more Congolese participants (n = 276, 53.8%) than Burundian (n = 236, 46.0%), with one participant reporting an unspecified nationality. Nearly half of the participants were illiterate (n = 236, 46.0%) with the most common level of education being primary school (n = 248, 48.3%) followed by a lack of formal education (n = 162, 31.6%). The vast majority of the 513 participants were Christian (n = 484, 94.3%), unemployed (n = 383, 74.6%), and single (n = 396, 77.1%).

### Health status and surgical pathology

The majority of participants self-reported generally good health (n = 386, 75.2%), but most also endorsed having an illness (n = 394, 76.8%) within the past year (Table 3). A high proportion reported using formal health services (i.e. hospital or clinics) in the camp within the past year (n = 495, 96.5%) but also sought a traditional healer for their concerns (n = 113, 22.0%). For self-perceived concerns amenable to surgical intervention, 152 patients (29.6%) had wounds, 85 (16.6%) had congenital deformities, 52 (10.1%) had burns, 52 (10.1%) had soft tissue pathology such as goiter and 172 (33.5%) reported or other pathologies (i.e lipomas, possible tumors, etc.). Most patients had only one issue (n = 374, 72.9%), while 139 (27.1%) had multiple concerns. Among all participants, 282 (55.0%) reported an ongoing or untreated problem. Families of 333 children (64.9%) sought care for all concerns while 40 families (7.8%) deferred care for at least one problem (i.e. sought care for one problem but did not seek care for another problem), and 140 (27.3%) did not seek care at all. Anatomical distribution of concerns included the face/head/neck (n = 247, 48.1%), extremities (n = 182, 35.5%), abdomen (n = 99, 19.3%), groin (n = 75, 14.6%), chest/breast (n = 33, 6.4%), and back (n = 25, 4.9%).

### Three Delays Model

**Figure 2:**
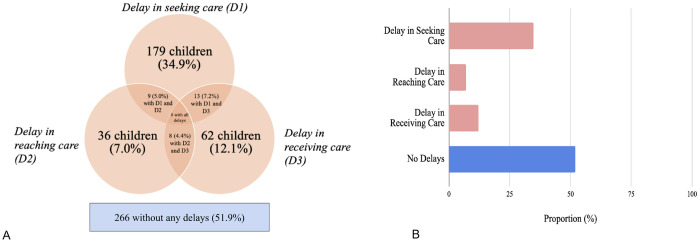
a. Prevalence of delays for not receiving surgery (n = 513). Note: D1, delay in seeking care; D2, delay in reaching care; D3, delay in receiving care b. Proportion of respondents with delays for not receiving surgery (n = 513)

Of 513 children with a condition potentially amenable to surgical intervention, 266 (51.9%) had no reported delays, while 247 (48.1%) experienced at least one type of delay for one or more of their concerns. Specifically, 179 children (34.9%) had a delay in seeking care (D1), while 36 children (7.0%) had a delay in reaching care (D2), and 9 had a delay in both. A total of 62 children (12.1%) had a delay in receiving care (D3), including 13 and 8 children who also experienced D1 and D2, respectively. There was no child with all three delays (Fig. 2a and 2b). A total of 64 (12.5%) children ultimately received minor or major surgery for at least one of their issues.

Among 139 (27.0%) participants with multiple concerns, different delays (i.e. delays 1 and 3) were experienced by 30 (21.6%) participants who had multiple problems while the rest (n = 109, 78.4%) experienced the same delay for all of their problems. For the 179 (34.9%) children who experienced delays in seeking care, the primary barriers included initially seeking care from a traditional healer (n = 50, 27.9%), a lack of perceived need (n = 23, 12.8%), and a perceived lack of available healthcare services (n = 15, 8.4%) (Table 4). There were 135 (75.4%) participants who were unable to identify a specific reason for the delay in seeking care, which may represent the complicated landscape of factors affecting health seeking behavior in refugee camp settings that the survey did not explicitly ask.

Regarding delays in reaching and receiving care (delays 2 and 3), most refugees reported total travel time to a health center of less than one hour (n = 464, 90.4%) with the next most common duration being one to two hours (n = 31, 6.3%). Most reported waiting for transportation for less than one hour (n = 484, 94.3%), with only 11 (2.1%) needing to wait more than one hour. Taken together, 36 (7.0%) children experienced a delay in reaching care. At the health center, the most common waiting period was 3–5 hours (n = 218, 42.5%); a majority of children waited more than two hours (n = 280, 54.6%), which constitutes a delay in receiving care as previously defined (Table 5). There were 18 (3.5%) children with no waiting times for transportation or travel times available, and 19 children (3.7%) without waiting times at the hospital before receiving care.

Characteristics that had a statistically significant association with delays in seeking surgical care (D1) included older age (children aged 0–5 and 6–11 years were less likely to delay seeking care than those aged 12–18 (aOR: 0.46; 95% CI 0.22–0.98, aOR: 0.38; 95% CI 0.20–0.68, respectively)), healthy self-perception (aOR: 2.22; 95% CI 1.31–3.86), no prior utilization of PHC services (aOR: 5.55; 95% CI 1.85-20.0), and burn injury pathology (aOR: 2.86; 95% CI 1.46, 5.70). The only characteristic with a statistically significant association with reaching care in a timely matter was pathology; those with miscellaneous pathology (aOR: 0.28; 95% CI 0.10–0.70) were less likely to experience a delay in reaching care (D2) relative to those with wounds. Congolese nationality (aOR: 3.39; 95% CI 1.76–6.85) was associated with delays in receiving care at the hospital (D3), as well as self-perception of being unhealthy (aOR: 1.97; 95% CI 1.02–3.75) (Table 6).

## Discussion

This research represents one of few efforts to rigorously assess barriers to receiving surgical care among pediatric patients in a humanitarian setting, particularly in sub-Saharan Africa. Our findings show that nearly half of children with a health concern that could be surgically intervenable experienced a delay at some point in the surgical care continuum, with most delays occurring in the initial stage: seeking care. Identifying specific characteristics associated with each delay can lead to more targeted interventions to reduce barriers to surgical care for children living in humanitarian settings.

Regarding delays in seeking care (D1), our analysis shows that 27.3% of children did not seek care for any of their potentially surgically-correctable concerns. Several factors were strongly associated with not seeking care at PHCs, including the initial consultation of traditional healers instead. A predilection among many families to first seek care from traditional healers may indicate that health facilities in the camp are perceived as inadequate or untrustworthy. This suggests that traditional healing may be preferred for reasons that were not captured in this analysis, such as convenience or perceived trustworthiness [21–23]. Of note, less than 10% of patients cited the unavailability of health services as a reason that they did not to seek care at a PHC, which may be a result of a lack of awareness about available health services or hearing anecdotes of delayed or suboptimal care experienced by friends and relatives [24].

Older children also had more delays in seeking care, potentially due to having additional responsibilities of working and/or caring for younger siblings, having more autonomy from parents in deciding whether to seek care, and/or holding negative perceptions of the camp’s healthcare system due to personal experiences for prior issues or those of family members or friends [25, 26]. Additionally, patients with wounds were more likely to seek care than patients with burns or other pathologies; this may be attributable to the stigmatizing and easily visible nature of wounds, prompting care-seeking at a health facility while preferentially seeing traditional healers for less acute pathologies [27, 28]. Given the humanitarian context of the camp, many families may have had lifelong limitations in accessing health care, making them less likely to seek care when ill. We found that patients who had not previously sought care at a PHC were less likely to seek care for potentially surgically-intervenable concerns.

These findings suggest that interventions focused on reducing stigma and building trust with households that have never previously used PHCs is likely an effective place to start. However, to reduce morbidity and mortality the quality and availability of services must be available to those who seek care —this will likely become increasingly challenging given the rapidly changing funding landscape for humanitarian health efforts in 2025. Recognizing the popularity of consulting traditional healers, other high-yield interventions could include collaborative partnerships between physicians and traditional healers to identify surgically amendable problems, to understand each other’s approach to healing, and to refer appropriate patients to PHCs when needed [29]. Doing so could be a first step towards integrating traditional healers into the biomedical health system and allowing physicians to provide more culturally-sensitive care [30].

Regarding delays in reaching care (D2), we found it encouraging that only 7.0% of participants reported being unable to reach care. This is likely because most healthcare services in the camp are free and readily accessible by foot. If transportation is required, it is often just a short distance, so financial concerns related to transportation are minimal. Communal collaboration also likely contributes to helping those in need reach the camp’s health facilities. Patients with wounds were more likely to have delays in reaching care relative to other pathologies, possibly due to the inability to mobilize independently. Interventions to address this could include implementation of a WhatsApp hotline to more promptly respond to patients with mobility issues and to provide basic treatment at home or to more rapidly triage high acuity transportation needs.

Regarding delays in receiving care (D3), 12.1% of all children experienced delays in receiving care at the camp’s health centers, which is significantly lower than the 42.9% reported by Concepcion et al in their 2020 piece analyzing delays in pediatric surgical care in Somaliland [15]. However, our results show that Congolese patients were more likely to experience delays in receiving care relative to Burundian patients.

This was surprising given that health services in the camp are intended to be free and accessible to all patients regardless of age, religion, gender, or nationality. Previous work from our group found that Congolese and Burundian patients had no significant demographic differences asides from education [16]. However, because Congolese refugees have been in the camp longer than Burundian patients, they may have previously sought care when the camp’s health infrastructure was more rudimentary, which may contribute to differing perceptions of stafing and quality of care, however we are unable to explore those views within our dataset. Language services are available for all patients regardless of nationality, but differences in cultural competency from hospital providers or staff may have contributed to the ethnic-based disparity in receiving care that we found on our analysis [31]. Interventions to address this ethnicity-based difference could include targeted patient navigation programs using CHWs [32]. Other confounding factors and systemic barriers should be explored to better understand differences in delays receiving care.

The barriers highlighted in this study likely represent challenges faced by refugee and underserved populations in other comparable settings. Preferentially seeking care from traditional healers instead of PHCs has been documented across various refugee and LMIC settings [33, 34]. This behavior is often driven by familiarity/trust in traditional practices, affordability, and perceived cultural alignment [35]. Interventions that bridge traditional and biomedical care, such as collaborative partnerships with healers, could be replicated to enhance timely access to surgical and medical care. Although transportation was not found to be a highly prevalent barrier in Nyarugusu, similar issues are common in other refugee and rural LMIC settings, where healthcare facilities are sparse and transportation is costly or unavailable [36, 37]. The proposed use of mobile health units and on-demand transport services is feasible with existing infrastructure in Nyarugusu and comparable settings. Delays in receiving care due to inadequate staffing, equipment, and social support reflect broader challenges in LMIC healthcare systems. While Congolese nationality appears to correlate with delays in receiving care in our study, similar cultural disparities in receiving care are often observed across ethnic or national groups in other refugee or LMIC populations [38–40]. This underscores the importance of disaggregated data collection and targeted interventions addressing specific community needs, a strategy applicable in diverse settings.

### Limitations

This study’s strengths include its high response rate, cluster-randomized design, and large pediatric sample size, but there are several limitations. Surveys were administered by community health workers (CHWs) trained to identify potential surgical health issues based on respondent-reported pathology (e.g., swelling, burns, wounds, disabilities, history of surgery) rather than thorough physical examinations or other diagnostic measures. These oral assessments, not conducted by licensed medical professionals, may have led to over- or underestimation of the prevalence of surgical conditions. Future studies could involve CHWs using mobile technology to consult surgeons to improve diagnostic accuracy. Additionally, while Kiswahili is a shared language in the refugee camp, some participants such as Burundian refugees may prefer Kirundi. To mitigate this, surveys were translated into Kiswahili, and CHWs fluent in Kirundi or other indigenous languages (e.g., Kibembe) were used in specific zones. However, this approach may have introduced biases, such as inconsistencies in translation or social desirability bias. Moreover, some households were unavailable for sampling due to repatriation and relocation. The study’s cross-sectional design precluded follow-up to determine whether children with ongoing issues ultimately sought and received treatment or if they encountered delays in the referral process to receive care outside of the refugee camp. Children with severe conditions may have died before being surveyed, potentially underestimating the surgical burden. Ultimately, a longitudinal prospective study is warranted to track delays and long-term outcomes among pediatric patients in Nyarugusu. Self-reported data also may have introduced recall bias, leading to possible over- or underestimation of surgical burden and factors contributing to delayed care. Additionally, many respondents (135) did not cite specific reasons for delays in care, which may suggest that a complicated milieu of factors—rather than one or several tangibly discrete reasons—may contribute to patients’ care seeking behavior and should be accounted for in future studies.

## Conclusion

Children living in Nyarugusu refugee camp in Tanzania experience a significant burden of unmet surgical needs complicated by multifactorial barriers to seeking, reaching, and obtaining surgical care. Future interventions targeting these factors, especially those aimed at building trust with patients and increasing the accessibility of health care services in camp will be essential to reducing barriers to care and to improving outcomes for pediatric refugee patients. Applying similar strategies in comparable settings across the globe will strengthen health systems that serve some of the world’s most vulnerable populations.

## Figures and Tables

**Table 1:** Definition of the Three Delays

Delay Type	Definition
**Delay in Seeking Care (D1)**	Not seeking biomedical care for any reason including: - Consultation of a traditional healer - Perceived lack of need - Fear or mistrust - Financial limitations - Lack of knowledge about available treatment - Lack of accessible health services - Time constraints
**Delay in Reaching Care (D2)**	- Taking more than an hour to be transported to an in-camp health facility and/or - Waiting more than an hour for transportation
**Delay in Receiving Care (D3)**	- Waiting more than six additional hours after reaching an in-camp health facility to receive care

**Table 2:** Demographic profile of the pediatric study population (n = 513)

	Characteristic	Total (n = 513)
**Age, median [IQR]**		11.0 [6.0–16.0]
**Age Bracket (years)**	0–5	137 (26.7)
	6–11	140 (27.3)
	12–18	236 (46.0)
**Gender**	Female	272 (53.0)
	Male	241 (47.0)
**Nationality**	Burundi	236 (46.0)
	Democratic Republic of the Congo	276 (53.8)
	Unknown	1 (0.2)
**Religion**	Christian	484 (94.3)
	Muslim	22 (4.3)
	Unknown	7 (1.4)
**Education**	No formal education	162 (31.6)
	Primary	248 (48.3)
	Secondary	101 (19.7)
	Unknown	2 (0.4)
**Literacy Status**	Yes	277 (54.0)
**Marital Status**	Single/Unmarried	396 (77.1)
	Married	19 (3.7)
	Divorce	1 (0.2)
	Unknown	97 (18.9)
**Occupation**	Small businesses	4 (0.8)
	Self-employment	3 (0.6)
	Housewife	6 (1.2)
	Farmer	5 (1.0)
	Unemployed	383 (74.6)
	Other	110 (21.4)
	Unknown	2 (0.4)
IQR interquartile range		

**Table 3:** Health status, surgical problems, and anatomical locations

	Characteristic	Total (n = 513)
**Self-perceived good health status**	Yes	386 (75.2)
**Prior use of Primary Health Center within past year**	Yes	495 (96.5)
**Illness within past year**	Yes	394 (76.8)
**Sought care from traditional healer**	Yes	106 (20.6)
**Pathology of Issue**	Wounds only	152 (29.6)
	Congenital deformities only	85 (16.6)
	Burns	52 (10.1)
	Soft-tissue growth/mass	52 (10.1)
	Other	172 (33.5)
**Number of Concerns**	1	374 (72.9)
	2	101 (19.7)
	3	22 (4.3)
	4	7 (1.3)
	5 or more	9 (1.7)
**Ongoing Concern**	Yes	282 (55.0)
**Anatomical region affected** ^a^		
	Head/Face/Neck	247 (48.1)
	Groin	75 (14.6)
	Breast/Chest	33 (6.4)
	Abdominal	99 (19.3)
	Extremity	182 (35.5)
	Back	25 (4.9)

a This variable represents the number and proportion of people who had at least one problem within the specified anatomical region out of a total of 513 participants. If a respondent had multiple problems within the same region, then the region was counted once.

**Table 4:** Reasons contributing to delays in seeking care

	Delay in seeking care (D1, n = 179 (%))^a^
No time	0 (0)
Fear/lack of trust	9 (5.0)
No perceived need	23 (12.8)
Sought traditional healthcare	50 (27.9)
No money for health care^b^	(0.6)
No money for transportation	1 (0.6)
Do not know where to find treatment	1(0.6)
Health care unavailable	15 (8.4)
No reason cited for a specific problem	135 (75.4)

a This variable was categorized by the number of participants who cited the listed reason for at least one specific concern. If the same concern applied to multiple problems, then it was counted once. The proportion was calculated by the total number of people with a delay in seeking care.

b Healthcare services in the camp are provided free of charge.

**Table 5:** Travel time and waiting time at the hospital

	Characteristic	Total (n = 513)
**Time traveling to health center**		
	Less than 1 h	464 (90.4)
	1–2 h	31 (6.3)
	Did not go to health center	18 (3.5)
**Time waiting for transportation**		
	Less than 1 h	484 (94.3)
	1–2 h	10 (1.9)
	3–5 hours	1 (0.2)
	Did not go to health center	18 (3.5)
**Time waiting to be seen at health center**		
	Less than 1 h	91 (17.7)
	1–2 h	123 (24.0)
	3–5 hours	218 (42.5)
	6–12 hours	51 (9.9)
	More than 12 hours	11 (2.1)
	Did not go to health center	19 (3.7)

**Table 6:** Multivariable analysis of factors associated with delays in seeking (D1), reaching (D2), and receiving (D3) surgical care among pediatric patients with surgical concerns in Nyarugusu refugee camp

	DELAY 1 (Seeking Care)			DELAY 2 (Reaching Care)			DELAY 3 (Receiving Care)		
Characteristic	Odds Ratio^a^	95% CI	p-value	Odds Ratio	95% CI	p-value	Odds Ratio	95% CI	p-value
**Age Group**									
12–18	REF	—	—	REF	—	—	REF	—	—
0–5	0.46	0.22, 0.98	0.044*	0.39	0.10, 1.46	0.2	1.01	0.35, 2.90	> 0.9
6–11	0.38	0.20, 0.68	0.001*	0.47	0.16, 1.28	0.2	0.96	0.43, 2.05	> 0.9
**Gender**									
Female	REF	—	—	REF	—	—	—	—	—
Male	1.22	0.82, 1.83	0.3	1.14	0.56, 2.33	0.7	0.67	0.37, 1.19	0.2
**Nationality**									
Burundi	REF	—	—	REF	—	—	REF	—	—
DRC	1.52	1.00, 2.34	0.053	1.5	0.70, 3.30	0.3	3.39	1.76, 6.85	< 0.001*
**Self-perceived healthy**									
Yes	2.22	1.31, 3.86	0.004*	0.66	0.29, 1.58	0.3	0.51	0.27, 0.98	0.04*
**Literacy Status**									
Yes	0.65	0.34, 1.22	0.2	0.73	0.25, 2.22	0.6	1.68	0.72, 4.10	0.2
**Illness in the past year**									
Yes	0.81	0.51, 1.29	0.4	1.73	0.69, 5.28	0.3	1.39	0.68, 3.09	0.4
**Pathology of Wound**									
Wounds only	REF	—	—	REF	—	—	REF	—	—
**Age Group**									
Congenital deformities only	1.36	0.70, 2.61	0.4	0.6	0.19, 1.65	0.3	1.04	0.40, 2.61	> 0.9
Burns	2.86	1.46, 5.70	0.002*	0.47	0.11, 1.52	0.3	0.36	0.08, 1.14	0.12
Swelling/Tumor	1.62	0.79, 3.27	0.2	0.32	0.05, 1.19	0.14	0.72	0.22, 1.96	0.5
Other	1.68	1.02, 2.77	0.042*	0.28	0.10, 0.70	0.009*	1	0.51, 1.99	> 0.9
**PHC Use**									
Yes	0.18	0.05, 0.54	0.003*	—	—	—	—	—	—

CI Confidence Interval; DRC Democratic Republic of the Congo; PHC Primary Health Center

a Odds ratios quantify the likelihood of experiencing a delay in seeking, reaching, or receiving care in one group relative to another, with values greater than 1 indicating higher odds of delay, values less than 1 indicating lower odds of delay, and a value of 1 suggesting no difference in delay.

* These p-values indicate statistical significance (p < 0.05)

## Data Availability

Data used and analyzed in the current study are not publicly available due to privacy and personally identifiable health information. De-identified, aggregate data are potentially available from the corresponding author upon request.
